# Monilethrix in Pattern Distribution in Siblings: Diagnosis by Trichoscopy

**DOI:** 10.4103/0974-7753.66918

**Published:** 2010

**Authors:** Nilam Jain, Uday Khopkar

**Affiliations:** Department of Dermatology, Seth G S Medical College and KEM Hospital, Mumbai, Maharashtra, India

**Keywords:** Androgenetic alopecia, hair shaft disorder, monilethrix, trichodermoscopy

## Abstract

Monilethrix is a heritable hair shaft defect characterized by localized or diffuse alopecia resulting from hair fragility over friction areas, predominantly the temporal and occipital regions, and follicular keratosis over the occipital region. However, it lacks macroscopic features that enable easy and rapid diagnosis in medical practice. Hair shaft microscopy is the basis for diagnosing monilethrix. We present a report of two Indian male siblings aged 24 and 21, who presented with thinning and hair loss from the scalp in male pattern distribution and multiple skin-colored follicular papules over the nape of the neck and bilateral forearms since childhood. Trichoscopy of scalp hair revealed characteristic uniform elliptical nodes and intermittent constrictions along with variation in hair shaft diameter, presence of few vellus hair and yellow dots, suggesting a diagnosis of monilethrix with early-onset androgenetic alopecia. Dermoscopy of the papules revealed multiple stubs of broken hair arising from them with a similar beaded appearance, suggesting a diagnosis of monilethrix. The diagnosis of monilethrix was confirmed with light microscopy and hair clipping. This report highlights the patterned distribution of hair loss in monilethrix probably due to the early unmasking of androgenetic alopecia and the use of trichoscopy as the diagnostic modality.

## INTRODUCTION

Dermatoscopy, also known as dermoscopy or epiluminescence microscopy, is the examination of skin lesions with a dermatoscope, a magnifier (typically ×10) with a light and liquid medium between the instrument and skin, thus illuminating without reflected light. Use of dermoscopy in hair disorders has been described in Alopecia Areata, androgenetic alopecia, trichotillomania and cicatricial alopecias, and is referred to as trichoscopy.[1–4] Diagnosis of hair shaft disorders is typically based on light microscopy and scanning electron microscopy.[3–6] Trichoscopic diagnosis in hair shaft disorders has been reported by a few authors in conditions like monilethrix, pili torti, pili trianguli and canaliculi, trichorrhexis nodosa, trichorrhexis invaginata and pili annulati.[3] We present here a case of two Indian siblings with monilethrix in whom trichoscopy aided rapid diagnosis.

## CASE REPORT

**Figures 1 and 2:**
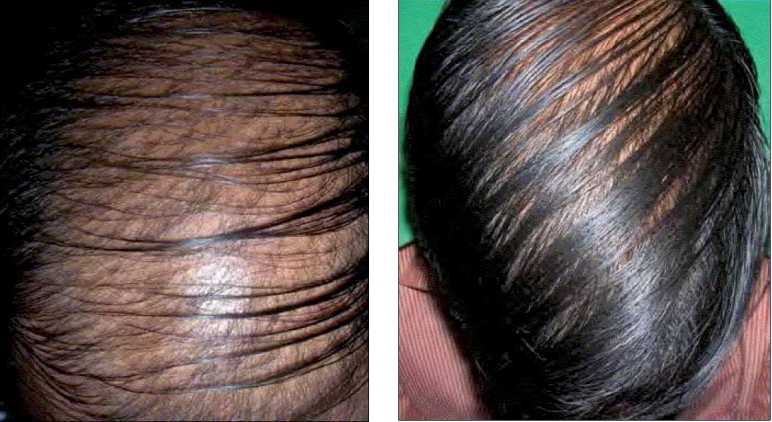
Diffuse hair loss with thinning of hair resembling androgenetic alopecia in siblings

**Figure 3:**
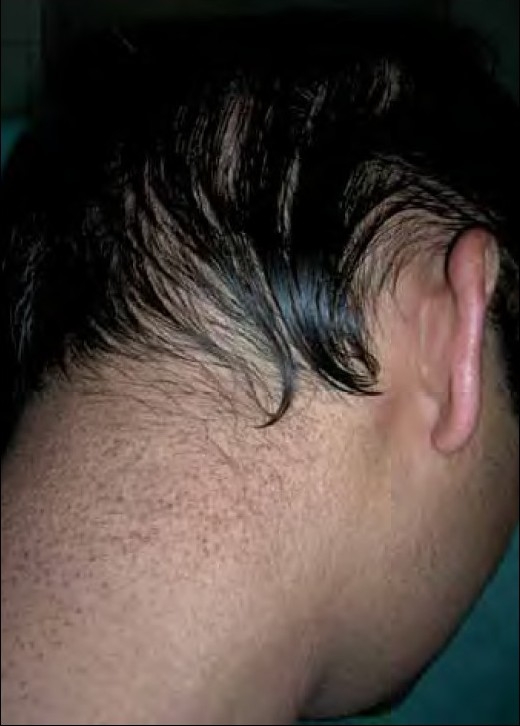
Multiple follicular keratotic papules over the nape of the neck and forearm

**Figure 4:**
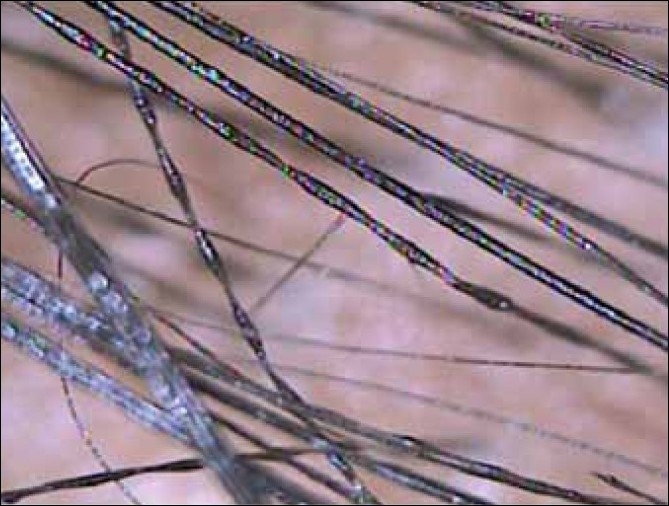
Beaded or moniliform appearance of the hair shaft with nodes and internodes

**Figure 5:**
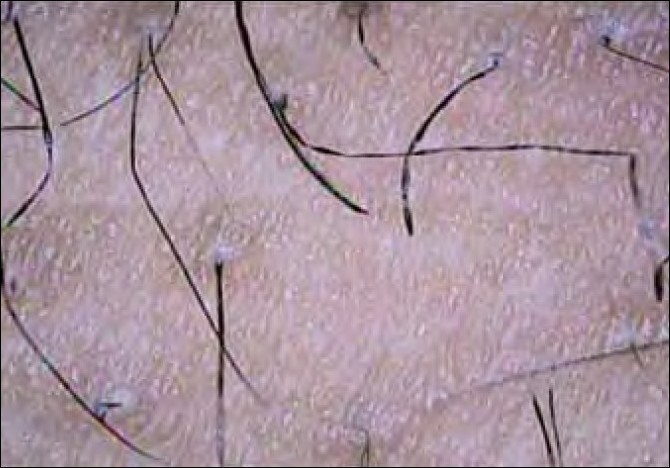
Hair arising from the keratotic papules shows internodal constrictions and regular bends

**Figure 6:**
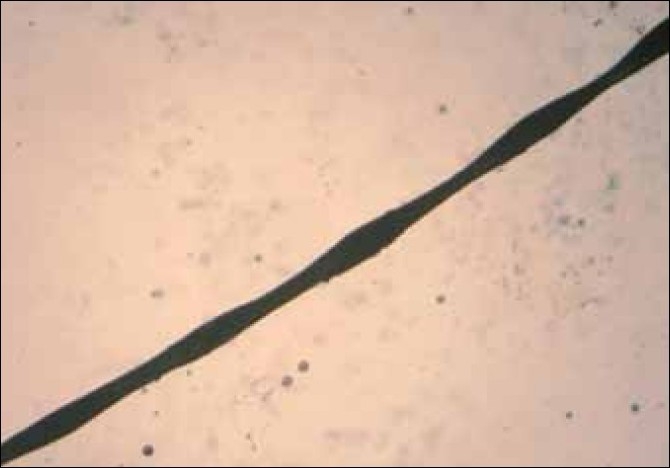
Light microscopy (20×) – beaded hair with constriction and fraying at internodal junctions

**Figure 7:**
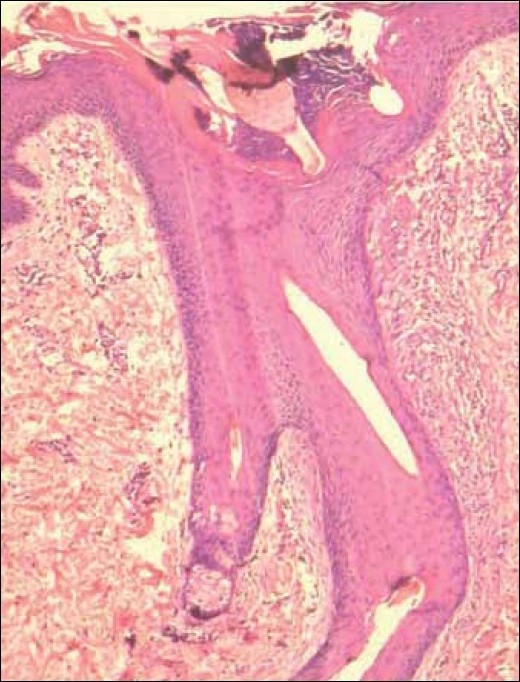
Abnormal hair with constriction, bulge and perifollicular fibroplasia and infiltrate in response to broken hair shaft within the follicle (scanner view)

Two siblings aged 24 and 21 years, born of non-consanguineous marriage, presented with progressive, diffuse hair loss and thinning of hair over the scalp since 14 years and 8 years, respectively. The complaints were not associated with seasonal variation or trauma to the hair. There was history of similar complaints in the father. Examination revealed sparse hair over the axillae and scalp involving the frontoparietal and temporal areas with relative sparing of the occipital area, a pattern seen in androgenetic alopecia [Figures 1 and 2]. Multiple keratotic papules were noted over the nape of the neck and the dorsal aspect of the forearms bilaterally [Figure 3]. Trichoscopy of the hairs over the occipital region and axillae revealed hair shafts having uniform elliptical nodes with intermittent constrictions and bent regularly at multiple locations [Figure 4] along with a majority of broken hair. Trichoscopy of the bald patches over the scalp showed some vellous hair along with mild variation in shaft thickness and yellow dots, suggestive of early androgenetic alopecia.[2,7] The hair shafts on the scalp showed a typical beaded appearance of moniletrhix on trichoscopy. The eyebrow hair was normal. Dermoscopy of the keratotic papules revealed short, brittle hair arising from them with similar features [Figure 5]. A diagnosis of monilethrix with early androgenetic alopecia was made based on these findings and confirmed with light microscopy, which revealed beaded hair with constriction and fraying at the internodal junction [Figure 6]. Scalp biopsy was not performed and trichogram was not possible due to marked fragility of hair. Histopathology of a keratotic papule revealed abnormal hair shaft with constriction and bulges associated with perifollicular fibroplasia and sparse lymphocytic infiltrate [Figure 7].[8]

## DISCUSSION

Monilethrix is a term of Greek and Latin derivation meaning “beaded hair.”[5,6] It is inherited in an autosomal-dominant manner, with variable expressivity. It has been mapped to the epithelial keratin gene cluster on 12q11–q13 and point mutations have been found in hair-specific keratins, especially hHb1 and hHb6.[1] Mutations have been found in desmoglein 4 in an autosomal-recessive form.[1,5] Affected individuals usually have normal appearing hair at birth but within the first few months of life, these fibers are replaced by short, fragile, brittle hair. Usually, the scalp is the only region involved, but the eyebrows and eyelashes may also be involved. Nails may show koilonychia.[3,5,6] Alopecia is more severe in areas prone to friction. Perifollicular erythema and follicular hyperkeratosis are commonly observed in the occipital region. Hair shaft microscopy shows hair fibers with regularly spaced elliptical, fusiform or spindle-shaped nodes of normal thickness separated by intermittent abnormal constrictions that are the sites of fracture.[1] The nodes have a diameter of normal hair and have a medulla whereas the internodes have no medulla. Pigment is present in both the segments. Scanning electron microscopy may reveal nodes with normal or worn transverse cuticular scales and internodes with dense longitudinal pattern of scales and ridging. Trichoscopy may reveal hair shafts with a beaded appearance, bent regularly at multiple locations, with a tendency to curve in different directions. These findings have been described as “regularly bended ribbon” sign by some authors.[1,3] Presence of these characteristic findings of moniletrhix with alopecia in male pattern distribution were the highlights of our case. Incidentally, on trichoscopy, additional findings of evolving androgenetic alopecia were noted. Hair fragility due to monilethrix may have resulted in early presentation or unmasking of androgenetic alopecia.

## References

[1] Rakowska A, Slowinska M, Czuwara J, Olszewska M, Rudnicka L (2007). Dermoscopy as a tool for rapid diagnosis of monilethrix. J Drugs Dermatol.

[2] Ross EK, Vincenzi C, Tosti A (2006). Videodermoscopy in the evaluation of hair and scalp disorders. J Am Acad Dermatol.

[3] Tosti A, Tosti A (2007). Hair shaft disorders. Dermoscopy of hair and scalp: Pathological and clinical correlation, illustrated edition.

[4] Liu CI, Hsu CH (2008). Rapid diagnosis of monilethrix using dermoscopy. Br J Dermatol.

[5] Sperling LC, Bolognia JL, Lorizzo JL, Rapini RP (2008). Alopecias. Dermatology.

[6] Paus R, Olsen EA, Messenger AG, Wolff K, Goldsmith LA, Katz SI, Gilchrist BA, Paller AS, Lefell DJ (2008). Hair growth disorders. Fitzpatrick’s Dermatology in General Medicine.

[7] De Lacharriere O, Deloche C, Misciali C, Piraccini BM, Vincenzi C, Bastien P (2001). Hair diameter diversity: A clinical sign reflecting the follicle miniaturization. Arch Dermatol.

[8] De Berker DA, Messenger AG, Sinclair RD, Burns T, Breathnach S, Cox N, Griffiths C (2004). Disorders of hair. Rook’s Textbook of Dermatology.

